# Addition of Spray-Dried Plasma in Phase 2 Diets for Weanling Pigs Improves Growth Performance, Reduces Diarrhea Incidence, and Decreases Mucosal Pro-Inflammatory Cytokines

**DOI:** 10.3390/ani14152210

**Published:** 2024-07-30

**Authors:** Hannah M. Bailey, Natalia S. Fanelli, Joy M. Campbell, Hans H. Stein

**Affiliations:** 1 Division of Nutritional Sciences, University of Illinois, Urbana, IL 61801, USAnataliad@illinois.edu (N.S.F.); 2 APC LLC, Ankeny, IA 50021, USA

**Keywords:** cytokines, growth performance, low crude protein, nursery pigs, spray-dried plasma

## Abstract

**Simple Summary:**

An experiment was conducted to test the hypothesis that pigs fed a low-protein diet containing 6% spray-dried plasma during phase 1 have improved growth performance and intestinal health if fed a low-protein diet during phase 2 containing spray-dried plasma. Low-protein phase 1 diets contained 0 or 6% spray-dried plasma, whereas low-protein, as well as normal-protein, phase-2 diets contained 0 or 2.5% spray-dried plasma. Including spray-dried plasma in low-protein diets improved growth performance during the initial 14 d post-weaning, but not during the combined phase 1 and phase 2 periods. Pigs fed a low-protein diet with spray-dried plasma in phase 1 and then a normal protein diet with spray-dried plasma in phase 2 had an improved growth performance compared with pigs fed a diet without spray-dried plasma, and the villus-height-to-crypt-depth ratio in the jejunum was reduced. Diarrhea and pro-inflammatory cytokines were lower in pigs fed diets with spray-dried plasma or diets low in protein compared with pigs fed diets without spray-dried plasma and with normal protein, indicating less intestinal inflammation.

**Abstract:**

The hypothesis that pigs fed a low crude protein (CP) diet with 6% spray-dried plasma (SDP) in phase 1 will have improved growth and intestinal health if the phase-2 diet contains 2.5% SDP was tested. Three hundred weaned pigs were used. Growth performance, feces, blood, and intestinal tissue were evaluated. Pigs fed 6% SDP in phase 1 had improved average daily gain (ADG) and final body weight (BW), but had reduced villus-height-to-crypt-depth ratio in phase 2 if 2.5% SDP was included in the normal-CP diet (*p* < 0.05), but not in the low-CP diet. Diarrhea incidence was less (*p* < 0.05) with 2.5% SDP in the phase 2 diet and for the low-CP diet. Ileal mucosa interleukin-1α (IL-1α) and IL-1β decreased (*p* < 0.05) for pigs fed the phase-1 diet with 6% SDP compared with pigs fed the diet without SDP. Addition of 2.5% SDP in phase 2 reduced (*p* < 0.05) IL-1β compared with the diet without SDP. Although the combination of SDP and low CP did not affect intestinal health in phase 2, diarrhea incidence and pro-inflammatory cytokines were reduced in pigs fed SDP in phase 1 or phase 2 or if a low-CP diet was fed.

## 1. Introduction

Spray-dried plasma (SDP) is included in diets fed to pigs during the initial 14 d post-weaning [1,2]; however, pigs fed a diet without SDP during the initial 7 d post-weaning have a greater improvement in feed intake and daily gain from d 8 to 14 post-weaning compared with pigs fed a diet with SDP in the phase 1 diet [2]. A lasting positive carryover effect of feeding SDP on growth performance is not always observed for pigs fed a diet with SDP longer than 14 d post-weaning [3], but improvements in the growth performance of pigs fed a diet with SDP are greater when pigs are under stress because of change in diet or insufficient nutrition [1,2]. Due to the reduced use of antibiotic growth promoters and zinc oxide in diets for newly weaned pigs, weanling pigs often experience increased dietary stress and more diarrhea. To alleviate this, the level of crude protein (CP) in diets for weanling pigs is routinely reduced [4], because the reduction in dietary CP in diets that do not contain antibiotic growth promoters is effective in reducing post-weaning diarrhea [4,5,6]. Feeding diets with reduced CP, although effective in reducing diarrhea, may result in feeding diets with amino acid (AA) concentrations below recommended levels. However, adequate provision of nutrients is vital during the post-weaning period, as pigs are exposed to greater pathogen loads, which can up-regulate the immune system, resulting in slightly greater AA requirements for the synthesis of cytokines and other immune cells [7]. However, inclusion of SDP in diets for newly weaned pigs can contribute to a mitigation of the intestinal challenges in pigs [8,9]. Spray-dried plasma is, therefore, often fed during the initial 7-to-14 d post-weaning, but in diets with reduced zinc oxide, it is possible that beneficial effects of SDP are obtained after this period. However, it is not known how the interaction between feeding low-CP diets and SDP in the phase-2 period affects pig growth performance and intestinal health. Therefore, this experiment was conducted to test the hypotheses that (1) pigs fed a low-CP diet containing 6% SDP during phase 1 of the nursery period continue to have increased growth performance and improved intestinal health if fed a low-CP diet during phase 2 compared with pigs fed a diet without SDP in phase 1; and (2) pigs fed a low-CP diet with 6% SDP during phase 1 have improved growth performance and intestinal health if the phase-2 diet is supplemented with 2.5% SDP.

## 2. Materials and Methods

The Institutional Animal Care and Use Committee at the University of Illinois reviewed and approved the protocol for this experiment. Pigs that were the offspring of Line 359 boars and Camborough females (Pig Improvement Company, Hendersonville, TN, USA) were used.

### 2.1. Diets, Animals, and Experimental Design

Spray-dried plasma (Appetein; bovine) was sourced from American Protein Corporation LLC, Ankeny, IA, USA. Seven diets were prepared (Table 1, Table 2 and Table 3). Two phase-1 diets with reduced CP were formulated with 8% soy protein concentrate (SPC) or with 6.0% SDP. Four phase 2 diets were formulated with low CP and with either 2.5% SPC or 2.5% SDP, or with normal CP and either 4.0% SPC or 2.5% SDP. The normal-CP diets were formulated according to the current recommendations [10], whereas the low-CP diets were formulated to contain 2-to-3 percentage units less CP than recommended [10]. A common phase-3 diet was formulated at a normal CP concentration without SDP. Vitamins and minerals were included in all diets to meet or exceed the current nutritional requirement estimates of weanling pigs [10]. No antibiotic growth promoters or other additives were included in the diets, and Zn and Cu were not included at pharmacological levels.

Three-hundred pigs were weaned at approximately 20 ± 2 d with an initial body weight, (BW) of 6.36 ± 0.78 kg and randomly allotted to an incomplete 2 × 2 × 2 factorial arrangement with 6 dietary treatments: (1) phase 1, 8% SPC and phase 2, 4% SPC with normal CP; (2) phase 1, 8% SPC and phase 2, 2.5% SPC with low CP; (3) phase 1, 6% SDP and phase 2, 4% SPC with normal CP; (4) phase 1, 6% SDP and phase 2, 2.5% SPC with low CP; (5) phase 1, 6% SDP and phase 2, 2.5% SDP with normal CP; and (6) phase 1, 6% SDP and phase 2, 2.5% SDP with low CP. There were 5 pens of barrows and 5 pens of gilts per dietary treatment with 5 pigs per pen for a total of 60 pens that were divided in 2 blocks where the blocking factor was weaning group. The two blocks were weaned two weeks apart and each group was weaned into an empty, cleaned, and disinfected room. Pens (1.2 × 1.4 m) had fully slatted plastic flooring and a feeder and a drinking nipple were installed in each pen. Dietary treatments were randomly allotted to pens throughout the room. Phase-1 diets were fed from d 1 to 14 post-weaning, phase 2 diets were fed from d 15 to 28 post-weaning, and the phase 3 common diet was fed from d 29 to 42. All pigs were allowed *ad libitum* access to feed and water throughout the experiment and all diets were provided in a meal form. 

Pigs were weaned from sows that are free of porcine respiratory disease and porcine, atrophic rhinitis, swine dysentery, and *Acintobacillus pleuropneumoniae*. Sows were vaccinated for *Lawsonia intracellularis*, parvo virus, and circovirus, but no vaccines were administered to the weaned pigs during the experiment. 

### 2.2. Sample Collection

Fecal scores were visually assessed every other day for 42 d by 2 independent observers, using a score from 1 to 5 (1 = normal feces; 2 = moist feces; 3 = mild diarrhea; 4 = severe diarrhea; and 5 = watery diarrhea). The average score of the two observers was calculated. Individual pig weights were recorded at the beginning of the experiment and at the end of each phase. Daily feed allotments were recorded and feed left in the feeders was weighed at the end of each phase. Data collected for pig weights and feed allowance were summarized to calculate average daily gain (ADG), average daily feed intake (ADFI), and gain-to-feed (G:F) ratio for each pen and dietary treatment group. Data were summarized for each phase and over the entire experiment.

At the beginning of the experiment, one pig in each pen with the BW closest to the pen average was identified, and 2 blood samples were collected on d 7, 14, and 28 from the jugular vein of this pig. Therefore, blood samples were collected from 5 barrows and 5 gilts per dietary treatment. One blood sample was collected in vacutainers with ethylenediaminetetraacetic acid (EDTA), and the other blood sample was collected in heparinized vacutainers. Blood samples were stored on ice immediately after collection, and EDTA blood samples were delivered to the University of Illinois Veterinary Diagnostic Laboratory for analysis of white blood cell, neutrophil, and lymphocyte cell counts in the whole blood. Following analysis, samples were centrifuged at 4000× *g* for 13 min to recover the plasma, which was stored at −20 °C until analysis for free AA. The blood collected in heparinized vacutainers was centrifuged at 4000× *g* for 13 min to recover the plasma, which was stored at −20 °C until analysis for plasma urea nitrogen, albumin, and total plasma protein using a Beckman Coulter Clinical Chemistry AU analyzer (Beckman Coulter, Inc., Brea, CA, USA). Globulin was calculated as the difference between total protein and albumin and the albumin/globulin ratio was calculated. At the end of phase 2 (d 28), the pig that was used for blood sampling was euthanized via captive bolt stunning and intestinal tissue and mucosa were collected.

### 2.3. Intestinal Morphology

Tissue samples from the jejunum were collected approximately 150 cm from the pylorus on d 28 for morphology analysis. Tissue samples were approximately 5 cm in length. All intestinal samples were opened longitudinally along the mesenteric attachment, rinsed with phosphate buffered saline, pinned serosa side down on a piece of cardboard [11], and then fixed by immersion in 10% neutral buffered formalin until analysis. After fixation, jejunum samples were sent to Veterinary Diagnostic Pathology, LLC (Fort Valley, VA, USA) where they were sectioned (5 mm thick cross-sections) and embedded in paraffin for slide preparation. For each sample, 3 to 4 transverse sections were stained with hematoxylin and eosin for histological analysis. Slides were then scanned using a 2.0 HT NanoZoomer (Hammatsu, Bridgewater, NJ, USA), and for each slide, 10 intact villi and the associated crypts were measured using NDP.View2 (Hammatsu, Bridgewater, NJ, USA). Villus height was measured from the villus tip to the base and crypt depth was measured from the crypt-villus junction to the base of the crypt with subsequent calculation of villus-height-to-crypt-depth ratio. Villus width and lamina propria width were measured at the midpoint of the villus. Villus width was measured at the third top of the villus and at the level of the crypt–villus junction to calculate villus surface area. 

### 2.4. Secretory Immunoglobulin A and Cytokine Analysis

Scrapings of jejunum and ileum mucosa were collected approximately 160 cm from the pylorus and 80 cm from the ileal-cecal junction, respectively. Mucosa samples were washed with phosphate buffered saline, snap frozen in liquid N, and stored at −80 °C until analysis. Intestinal mucosa samples were homogenized in phosphate buffered saline containing protease inhibitors (SKU, P8340; Sigma-Aldrich, St. Louis, MO, USA). The supernatant was collected and used for analysis of secretory immunoglobulin A (sIgA) using an enzyme linked immunosorbent assay (ELISA) kit according to the manufacturer’s recommended procedures (Bethyl Laboratories, Inc., Montgomery, TX, USA). Concentrations of sIgA were expressed on a per mg protein basis and all values were normalized with total protein concentration in each sample quantified by a Pierce bicinchoninic acid Protein Assay Kit (Thermo Scientific, Woltham, MA, USA). The supernatant was also used to determine concentrations of the following cytokines: interleukin-1α (IL-1α), IL-1β, IL-1 receptor antagonist, IL-2, IL-4, IL-6, IL-8, IL-10, IL-12, and IL-18, using a MILLIPLEX MAP kit (MilliporeSigma, Burlington, MA, USA) in a MAGPIX instrument with ProcartaPlex- multiplex technology (R&D Systems, Inc., Minneapolis, MN, USA).

### 2.5. Chemical Analysis

All diet and ingredient samples were analyzed in duplicate for concentrations of gross energy, using an isoperibol bomb calorimeter (Model 6400, Parr Instruments, Moline, IL, USA), and N by combustion (method 990.03; [12]), using a LECO FP628 analyzer (LECO Corp., Saint Joseph, MI, USA), with the subsequent calculation of crude protein as N × 6.25. Dry matter was also analyzed in diet and ingredient samples by oven-drying at 135 °C for 2 h (method 930.15, [12]), and these samples were also analyzed for dry ash (method 942.05; [12]). Minerals (i.e., Ca, P, Na, K, and Mg) were analyzed in diets and ingredients using inductively coupled plasma–optical emissions spectrometry (Avio 200, PerkinElmer, Waltham, MA, USA). Sample preparation included dry ashing at 600 °C for 4 h (method 985.01 A, B, and C; [12]) and wet digestion with nitric acid (method 3050 B; U.S. [13]). All diet and ingredient samples were analyzed for acid hydrolyzed ether extract using the acid hydrolysis filter-bag technique (Ankom^HCl^ Hydrolysis System; Ankom Technology, Macedon, NY, USA), followed by crude fat extraction using petroleum ether (Ankom^XT15^ Extractor; Ankom Technology, Macedon, NY, USA). All diet and ingredient samples were analyzed for AA (method 982.30 E (a, b, c); [12]). and diets were analyzed for total starch using the glucoamylase procedure (method 979.10; [12]).

### 2.6. Statistical Analysis

Normality of residuals were verified, and outliers were identified using the UNIVARIATE and BOXPLOT procedures, respectively (SAS Inst. Inc., Cary, NC, USA). Outliers were removed if they were located outside of the lower and upper far fences, which are located at 3× the interquartile range [14]. Data for mucosa inflammation were log2 transformed before statistical analysis to obtain normal distribution. Data for growth performance, diarrhea scores, tissue morphology, and mucosa inflammation were analyzed by ANOVA, using the PROC MIXED procedure of SAS (SAS Inst. Inc., Cary, NC, USA) in a randomized complete block design, with weaning group as the blocking factor and the pen as the experimental unit. The model included the fixed effect of dietary treatment and the random effects of block and replicate within block. The experimental design was an incomplete 2 × 2 × 2 factorial, but due to the incomplete factorial, contrast statements were used to determine effects of inclusion of SDP in phase-1 and phase-2 diets and level of dietary CP in phase 2. Contrasts included (1) interaction within phase-1 diets with SDP: phase-2 SDP × phase-2 CP; (2) interaction within phase-2 diets without SDP: phase-1 SDP × phase-2 CP; (3) main effect of phase-1 SDP; (4) main effect of phase-2 SDP; and (5) main effect of phase-2 CP. Blood samples were collected from the same pig each collection day; therefore, data were analyzed as repeated measures with unstructured variance based on the likelihood ratio test using the PROC MIXED and REPEATED procedures of SAS. The model included the fixed effects of dietary treatment and day and the interaction between dietary treatment and day. The pig was the experimental unit. The interaction between dietary treatment and day was not significant; therefore, contrast statements were used with coefficients for unequally spaced treatments being generated using the PROC IML statement in SAS to determine linear and quadratic effects of day on blood variables. Treatment means were calculated using the LSMEANS statement, and if significant, means were separated using the PDIFF option in the PROC MIXED procedure. Statistical significance and tendencies were considered at *p* < 0.05 and 0.05 ≤ *p* < 0.10, respectively.

## 3. Results

### 3.1. Growth Performance and Diarrhea Scores

All pigs remained healthy throughout the experiment. There was no difference in initial BW of pigs among treatments (Table 4), but at the end of phase 1, pigs fed the diet with 6% SDP had a greater (*p* < 0.01) BW, and ADG, ADFI, and G:F were greater during the initial 14 d post-weaning for pigs fed the diet containing 6% SDP compared with pigs fed the diet without SDP. Pigs fed the phase 1 diet with 6% SDP had greater ADFI and ADG during phase 2 and greater BW at the end of phase 2 if 2.5% SDP was included in the normal-CP phase 2 diet than if the normal-CP diet was without SDP, but inclusion of 2.5% SDP in low-CP diets did not influence ADFI, ADG, or BW in phase 2 (interaction, *p* < 0.10 for ADFI and *p* < 0.05 for ADG and BW). Pigs that were fed the phase 1 diet without SDP had greater ADG in phase 2 if diets had normal concentration of CP than low concentration of CP, but if pigs were fed the phase 1 diet with 6% SDP, dietary CP concentration in phase 2 did not affect ADG (interaction, *p* < 0.10). Inclusion of SDP in phase 1 or phase 2 diets did not influence the G:F in phase 2, but pigs fed the diet with normal CP had a greater (*p* < 0.01) G:F than pigs fed the low-CP diet.

In phase 3, where all pigs were fed the common diet without SDP, the ADG of pigs that were fed the phase 1 diet with 6% SDP was greater if fed the low-CP phase 2 diet without SDP than the normal-CP diet without SDP, but the ADG during phase 3 was not influenced by CP concentration in phase 2 if 2.5% SDP was included in the phase 2 diet (interaction, *p* < 0.10). In contrast, the ADFI during phase 3 and final BW of pigs fed the phase 1 diet with 6% SDP was not influenced by dietary CP in phase 2, but pigs fed the normal-CP diet had a greater ADFI and final BW if 2.5% SDP was also included in the diet than if the phase 2 diet did not contain SDP (interaction, *p* < 0.10 for ADFI and *p* < 0.05 for BW). The G:F ratio of pigs in phase 3 was not influenced by SDP inclusion in the phase 1 diet, but the G:F ratio increased (*p* < 0.10) for pigs fed the phase 2 diet without SDP compared with pigs fed the diet with SDP, and the G:F ratio in phase 3 also increased (*p* < 0.05) for pigs fed the low-CP diet compared with pigs fed the normal-CP diet. 

The overall ADG and ADFI from phase 1 to phase 3 was greater for pigs fed the phase 1 diet with 6% SDP if 2.5% SDP was included in the normal-CP diet in phase 2 compared with the normal-CP diet without SDP, but this was not the case if 2.5% SDP was included in the low-CP diet in phase 2 (interaction, *p* < 0.05). The overall ADFI tended to be greater for pigs fed the phase 1 diet with 6% SDP if pigs were fed the phase-2 low-CP diet without SDP compared with pigs fed the phase-1 diet without SDP and the phase-2 low-CP diet without SDP, but the inclusion of SDP in phase 1 did not affect ADFI if phase-2 diets without SDP had normal CP concentration (interaction, *p* < 0.10). Overall the G:F ratio of pigs was not influenced by inclusion of SDP in the phase-1 diet, but pigs fed the phase-2 diet with SDP had decreased (*p* < 0.05) overall G:F compared with pigs fed the phase-2 diet without SDP, and pigs fed the normal-CP diet in phase 2 had a greater (*p* < 0.05) overall G:F ratio than pigs fed the low-CP diet in phase 2.

Diarrhea scores during the initial 6 d post-weaning were reduced (*p* < 0.10) for pigs fed the diet with 6% SDP compared with pigs fed the diet without SDP (Table 5). During phase 2 (d 16 to 28), diarrhea scores were reduced (*p* < 0.05) for pigs fed the diet with 2.5% SDP compared with pigs fed the diet without SDP, and diarrhea scores were less (*p* < 0.01) for pigs fed the low-CP diet than for pigs fed the normal-CP diet. During phase 3 (d 30 to 42), pigs previously fed the phase 1 diet with 6% SDP had reduced (*p* < 0.05) diarrhea scores compared with pigs previously fed the phase 1 diet without SDP, and diarrhea scores were also reduced (*p* < 0.05) for pigs previously fed the phase 2 diet with 2.5% SDP compared with pigs fed the phase 2 diet without SDP.

### 3.2. Tissue Morphology

The villus height, villus width, and lamina propria thickness of the jejunum on d 28 were not influenced by phase 1 or phase2 inclusion of SDP or phase 2 dietary CP (Table 6). Pigs that were fed the phase 1 diet with 6% SDP had greater crypt depth in the jejunum if fed the normal-CP phase 2 diet with 2.5% SDP than if pigs were fed the normal-CP phase 2 diet without SDP, but the inclusion of 2.5% SDP in the low-CP phase 2 diet did not influence crypt depth in the jejunum (interaction, *p* < 0.01). Pigs that were fed the phase 1 diet with 6% SDP had decreased villus-height-to-crypt-depth ratio in the jejunum if 2.5% SDP was included in the normal-CP diet compared with the normal-CP diet without SDP, but villus-height-to-crypt-depth ratio was not influenced by the addition of 2.5% SDP to the low-CP phase-2 diet (interaction, *p* < 0.05). Pigs fed the phase-1 diet with 6% SDP had greater villus-height-to-crypt-depth ratio in the jejunum if fed the normal-CP phase 2 diet without SDP than the low-CP diet without SDP, but if pigs were fed the phase 1 diet with 6% SDP, CP concentration in the phase 2 diet without SDP did not affect villus-height-to-crypt-depth ratio (interaction, *p* < 0.05). Villus surface area in the jejunum was greater (*p* < 0.10) for pigs fed the phase 1 diet with 6% SDP than the phase 1 diet without SDP, but villus surface area was not influenced by inclusion of SDP or dietary CP in phase 2.

### 3.3. Secretory Immunoglobulin A and Mucosal Cytokines

*Jejunum:* Secretory IgA concentration in the jejunal mucosa on d 28 was not influenced by SDP inclusion in phase 1 or phase 2 or by dietary CP in phase 2 (Table 7). Pigs that were fed the phase 1 diet with 6% SDP had greater jejunal mucosa IL-10 on d 28 if fed the normal-CP phase 2 diet than if pigs were fed the low-CP phase 2 diet, but if pigs were fed the phase 1 diet without SDP, no impact of CP concentration in phase 2 was observed for IL-10 (interaction, *p* < 0.10). Mucosal concentrations of IL-1α and IL-18 in the jejunum were greater (*p* < 0.10) for pigs fed the normal-CP diet in phase 2 than pigs fed the low-CP diet, regardless of SDP inclusion in phase 2. The mucosal concentration of IL-4 was not influenced by dietary CP in phase 2, but it was greater (*p* < 0.05) if pigs were fed the phase-1 diet with 6% SDP than if fed the phase-1 diet without SDP, and the IL-4 concentration was greater (*p* < 0.05) for pigs fed the phase 2 diet with 2.5% SDP compared with pigs fed the phase 2 diet without SDP. 

*Ileum:* Pigs fed the phase 1 diet with 6% SDP had greater concentration of sIgA in the ileal mucosa if fed the normal-CP phase 2 diet without SDP compared with pigs fed the low-CP diet without SDP, but sIgA did not differ between pigs fed the normal or low-CP phase 2 diet if 2.5% SDP was included (interaction, *p* < 0.05). If pigs were fed the phase 1 diet without SDP, IL-10 in ileal mucosa was greater for pigs fed the low-CP diet without SDP in phase 2 compared with pigs fed the normal-CP diet without SDP, but dietary CP in phase 2 did not affect mucosal IL-10 concentration if pigs were fed the phase 1 diet with 6% SDP (interaction, *p* < 0.05). If pigs had been fed the phase 1 diet with 6% SDP, ileal mucosa concentration of IL-18 tended to increase if the normal-CP diet without SDP was fed in phase 2 compared with pigs fed the low-CP diet without SDP in phase 2, but IL-18 was not influenced by dietary CP in phase 2 if pigs had been fed the phase-1 diet without SDP (interaction, *p* < 0.10). The concentration of IL-1α and IL-1β in the ileal mucosa decreased (*p* < 0.05) and IL-6 and IL-12 tended to decrease (*p* < 0.10) if pigs were fed the phase 1 diet with 6% SDP compared with pigs fed the phase 1 diet without SDP. Likewise, pigs fed the phase 2 diet with 2.5% SDP had decreased (*p* < 0.05) concentration of IL-1β, but tended to have increased (*p* < 0.10) concentration of IL-4, in the ileal mucosa compared with pigs fed the phase 2 diet without SDP, regardless of dietary CP in phase 2 or SDP inclusion in phase 1. Pigs fed the normal-CP diet in phase 2 had greater (*p* < 0.01) mucosal IL-8 concentration compared with pigs fed the low-CP diet in phase 2, regardless of SDP inclusion in the phase 1 or phase 2 diet.

### 3.4. Blood Parameters

The concentration of white blood cells, neutrophils, and lymphocytes and the albumin-to-globulin ratio from d 7 to 28 was not influenced by inclusion of SDP in phase 1 or phase 2 diets or by dietary CP in phase 2 (Table 8). If pigs had been fed the phase 1 diet without SDP, albumin concentration in plasma from d 7 to 28 had a tendency to increase if the normal-CP diet without SDP was fed in phase 2 compared with pigs fed the low-CP diet without SDP, but albumin was not influenced by dietary CP in phase 2 if pigs had been fed the phase 1 diet with 6% SDP (interaction, *p* < 0.10). The concentration of plasma urea N from d 7 to 28 was less (*p* < 0.05) for pigs fed the phase 1 diet with 6% SDP compared with pigs fed the phase 1 diet without SDP. Total protein concentration in plasma of pigs was not influenced by the inclusion of SDP in phase 1 or phase 2 diets, but total protein was increased (*p* < 0.01) for pigs fed the normal-CP diet compared with pigs fed the low-CP diet in phase 2. Blood concentrations of white blood cells and neutrophils increased and then decreased from d 7 to 28 (quadratic, *p* < 0.05), whereas concentrations of lymphocytes, plasma urea N, albumin, and total protein, and the albumin-to-globulin ratio decreased and then increased from d 7 to 28 (quadratic, *p* < 0.05).

For pigs fed the phase 1 diet with 6% SDP, plasma Met tended to decrease from d 7 to 28 if pigs were fed the normal-CP phase 2 diet with 2.5% SDP compared with pigs fed the normal-CP phase 2 diet without SDP (Table 9), but plasma Met tended to increase for pigs if 2.5% SDP was included in the low-CP diet compared with pigs fed the low-CP diet without SDP (interaction, *p* < 0.10). Pigs fed the phase 1 diet with 6% SDP had increased (*p* < 0.01) concentrations of plasma Lys, Trp, Val, and Tyr and increased (*p* < 0.05) Gln and Ser from d 7 to 28 compared with pigs fed the phase 1 diet without SDP, regardless of dietary treatment in phase 2. In contrast, plasma Ile decreased (*p* < 0.01) from d 7 to 28 for pigs fed the phase 1 diet with 6% SDP compared with pigs fed the phase 1 diet without SDP, and plasma Ile was also less (*p* < 0.01) for pigs fed the phase 2 diet with 2.5% SDP compared with pigs fed the phase 2 diet without SDP. Pigs fed the normal-CP diet in phase 2 had decreased (*p* < 0.10) concentration of plasma Asn from d 7 to 28 compared with pigs fed the low-CP diet, whereas the concentration of ornithine was greater (*p* < 0.10) for pigs fed the normal-CP diet compared with pigs fed the low-CP diet in phase 2, regardless of SDP inclusion in phase 1 or phase 2. The concentration of most plasma AA, except for His, Met, Asp, and Cys, increased (quadratic, *p* < 0.05) for pigs from d 7 to d 28.

## 4. Discussion

An incomplete 2 × 2 × 2 factorial design was used in this experiment because phase 1 diets that were not low in CP were not used. The reason for this is that the use of low-CP diets in phase-1 diets is well documented and widespread [4,9,15], and the objective was, therefore, not to demonstrate the positive effects on diarrhea reduction in phase 1 by reducing dietary CP. As a consequence, only six diets were used in the experiment because the objective was to determine if SDP in low-CP phase 1 diets could provide health and performance benefits and if continuing to add SDP to phase 2 diets with low or normal CP has additional advantages.

Weaned pigs often face production challenges due to changes in environment, diet, and social structure. These changes can activate the immune response, leading to inflammation throughout the intestinal tract, post-weaning diarrhea, reduced feed intake, and decreased growth; thus, the subsequent development of the pig is negatively affected [16]. High-quality ingredients are used in post-weaning diets to ameliorate the stress caused by weaning, and SDP is included in post-weaning diets to improve pig growth performance and reduce intestinal inflammation [1,3,8]. Dietary SDP contributes to pigs maintaining immune homeostasis in the intestinal tract by preventing pathogen colonization in the mucosa [4,17], thereby reducing post-weaning diarrhea [4]. Reducing the concentration of CP in post-weaning diets by 2-to-3 percentage units below NRC requirements can also decrease the prevalence of diarrhea by reducing excess N fermentation in the large intestine [9,15]. The combination of low dietary CP and inclusion of SDP in the diet has not previously been tested, but it was hypothesized that this combination may have a complementary effect on diarrhea prevention due to the different modes of action of these strategies. The observation that inclusion of SDP in the low-CP diet decreased diarrhea incidence during the initial 6 d post-weaning is in agreement with Heo et al. [18] and Peace et al. [8], who reported that a low-CP diet or a diet with SDP is effective in reducing diarrhea during the initial wk post-weaning. In wk 3 and 4 post-weaning of the current experiment, continuing to feed a diet with SDP or low CP was also effective in decreasing diarrhea scores, which is in contrast with Le Bellego and Noblet [19], who reported that feeding reduced CP diets for 5 wk post-weaning did not affect the occurrence of diarrhea. Diets with low CP are generally fed to pigs in the initial first or second wk post-weaning, when they are most susceptible to diarrhea, but continuing to feed low-CP diets for more than 1 or 2 wk post-weaning may lead to losses in growth performance [5,9], as also observed in the present experiment.

The impact of feeding a diet containing 2-to-3 percentage units less CP than required on ADG, ADFI, and G:F of pigs is generally the greatest concern for producers [20]. The results of several experiments indicate that growth performance of pigs was unchanged or improved if pigs were fed low-CP diets [18,19,21]; however, reductions in dietary CP in diets for weanling pigs can negatively affect ADFI, feed efficiency, or final BW of pigs [22,23,24]. 

Inclusion of SDP in diets has consistently resulted in improved growth performance of weanling pigs [1,2], which was also observed in the current experiment when SDP was included in phase 1 and phase 2 diets. Inclusion of SDP in a low-CP diet may, therefore, ameliorate reductions in growth performance when feeding diets low in CP for longer periods post-weaning. However, the observation that ADG, ADFI, and d 28 BW were increased when SDP was included in the normal-CP diet, but not if SDP was included in the low-CP diet, indicates that the inclusion of SDP did not improve the growth performance of pigs fed a low-CP phase-2 diet, and the second hypothesis was, therefore, partly rejected. Crystalline Lys, Met, and Thr were supplemented to the low-CP diet in the current experiment, but decreased growth performance has been reported for pigs fed low-CP diets supplemented with these AA [24]. In contrast, supplementing branched-chain AA to low-CP diets increased feed intake and protein deposition in the muscle of pigs [25,26], indicating that branched-chain AA may be next limiting in low-CP diets [9]. Indispensable AA were reduced by 15% in the low-CP diets compared with the requirement [10] in the current experiment, and the reduced growth performance of pigs fed the low-CP diet indicates that dietary AA were insufficient to support maximum growth of the pig [27]. 

The reduction in the G:F ratio observed for pigs fed low-CP diets in phase 2 compared with pigs fed greater CP concentrations agrees with previous data [23], but reductions in growth performance can be compensated by improved efficiency in the subsequent phases [4,5,15], which was also observed in the current experiment. Pigs adapt to reduced feed intake immediately after weaning, and once they have adapted, protein turnover increases leading to compensatory growth [28]. Data from the current experiment indicate that pigs fed either the low-CP diet or a diet without SDP in phase 2 exhibited compensatory growth when all pigs were on the same plane of nutrition in phase 3. However, the final BW was greatest (1.51 kg heavier) for pigs fed both phase-1 and phase-2 diets with SDP compared with pigs fed a diet with normal CP, but without SDP in phase 2, indicating that supplementation with SDP for longer periods can further improve pig BW, which is in agreement with previous data [2]. However, as SDP is included in diets for pigs up to 40 d post-weaning, improvements in ADG, ADFI, or feed efficiency tend to be less significant [2].

Intestinal morphology may be maintained if dietary CP is reduced by up to 3 percentage units of NRC (2012) [10] recommended CP concentration [20]; but reducing dietary CP without AA supplementation may be associated with reduced villus height and crypt depth due to an insufficient supply of AA required to maintain the structure of the intestinal epithelium [9,29]. The observation that pigs fed low-CP diets had a reduced villus-height-to-crypt-depth ratio is in agreement with Chen et al. [30], whereas reducing dietary CP from 21 to 19% increased the villus-height-to-crypt-depth ratio [31]. The observation that inclusion of 6% SDP in the low-CP diet resulted in the villus-height-to-crypt-depth ratio not being different from that of pigs fed the normal-CP diet without SDP indicated that inclusion of SDP in a low-CP diet may maintain the intestinal barrier. Dietary SDP can increase villus height and decrease crypt depth in the intestine resulting in a greater villus-height-to-crypt-depth ratio compared with pigs fed a diet without SDP [32]. The greater crypt depth observed for pigs fed the normal-CP diet with SDP compared with pigs fed the diet without SDP is not in agreement with data demonstrating that there is no effect of dietary SDP on crypt depth throughout the small intestine [33,34,35,36]. A deeper crypt can indicate poorer performance [37], whereas a shallower crypt may imply less rapid cell turnover for villus renewal [38]. Therefore, the ratio of villus height to crypt depth is a more important parameter to evaluate intestinal barrier function than the crypt depth by itself [39].

Activation of the immune system may increase AA requirements, but generally protein deposition is reduced, and AA are used for the synthesis of immune cells rather than for growth of the pig [40]. Therefore, the improved growth performance observed for pigs fed diets with SDP indicates reduced immune cell synthesis. The observed decrease in mucosal pro-inflammatory cytokines IL-1α, IL-1β, IL-6, and IL-12 in the ileum of pigs fed SDP in phase 1 is in agreement with previous data indicating dietary SDP results in pigs maintaining intestinal mucosa immune homeostasis by suppressing synthesis of pro-inflammatory cytokines [41,42]. The pro-inflammatory cytokine IL-1β is responsible for priming and amplifying the subsequent intestinal immune response [43]. As a consequence, the reduction in IL-1β in the ileum for pigs fed SDP in both phase 1 and phase 2 indicates a lasting positive effect of dietary SDP on intestinal inflammation. No detrimental or beneficial complementary effects were observed between dietary SDP and low CP, but the pro-inflammatory cytokines IL-1α and IL-18 in the jejunum and IL-8 in the ileum decreased for pigs fed low-CP diets, which is in agreement with previous data [5,6]. Reduced pro-inflammatory cytokines synthesized in the mucosa throughout the small intestine indicates reduced intestinal inflammation, thereby contributing to reduced incidence of diarrhea in pigs fed a diet supplemented with SDP or a diet low in CP [5].

Plasma urea N is an indicator of protein utilization due to the positive correlation between plasma urea N and N excretion in the urine [44]. Therefore, the observed decrease in plasma urea N on d 7, 14, and 28 when 6% SDP was included in the diet from d 1 to 14 indicates improved protein utilization, which may have contributed to the better growth performance of pigs fed diets containing SDP. This observation is in agreement with previous data [45,46,47]. The observation that pigs fed diets with low CP had decreased albumin in the plasma from d 7 to 28 is in agreement with Limbach et al. [5] and likely reflects a reduced need to transport AA in these pigs due to reduced absorption. However, the observed reduction in albumin may have contributed to the reduced growth performance observed for pigs fed the low-CP diet, because albumin binds and transports nutrients in the blood [48,49]. The increased concentration of most AA that was observed from d 7 to d 28 is most likely a reflection of the greater feed intake on d 28 and, therefore, more AA being absorbed compared with d 7. The observation that plasma concentrations of Lys, Trp, and Val were greater in phase 2 if pigs were fed the diet containing SDP in phase 1 may be a result of improved intestinal health that allowed for a more efficient absorption of AA when pigs had been fed SDP in phase 1. 

## 5. Conclusions 

The inclusion of 6% SDP in diets containing 2-to-3 percentage units less CP than required during the initial 14 days post-weaning improved growth performance, but the inclusion of SDP in low-CP diets fed to pigs until 4 wk post-weaning did not improve growth performance parameters. However, supplementing low-CP phase 1 and normal-CP phase 2 diets with SDP resulted in greater BW of pigs on d 42 post-weaning. The combination of reducing dietary CP by 2-to-3 percentage units and inclusion of SDP in the diet did not affect diarrhea incidence of pigs, but dietary SDP or diets low in CP were effective in reducing diarrhea 3-to-4 wk post-weaning. This may be associated with decreased mucosal pro-inflammatory cytokine synthesis throughout the intestine of pigs fed diets with SDP or low in CP, indicating decreased intestinal inflammation.

## Figures and Tables

**Table 1:** Ingredient composition of experimental diets (as-fed basis) ^1^.

Item, %	Phase 1		Phase 2				
	Low Crude Protein		Low Crude Protein		Normal Crude Protein		Phase 3
Spray-Dried Plasma, %:	–	6.0	–	2.5	–	2.5	–
Spray-dried plasma	–	6.00	–	2.50	–	2.50	–
Corn, ground	52.85	53.71	59.14	59.28	49.70	51.22	62.50
Soybean meal, 46% crude protein	12.70	14.20	22.00	22.00	25.00	25.00	32.00
Whey powder, dried	20.00	20.00	10.00	10.00	15.00	15.00	–
Soy protein concentrate	8.00	–	2.50	–	4.00	–	–
Soybean oil	3.10	3.10	3.10	3.10	3.10	3.10	2.50
Limestone, ground	0.91	1.17	0.90	1.03	0.93	1.05	1.00
Dicalcium phosphate	1.20	0.88	1.30	1.15	1.07	0.95	0.90
Sodium chloride	0.40	0.40	0.40	0.40	0.40	0.40	0.40
L-Lys HCl	0.40	0.25	0.36	0.28	0.41	0.40	0.35
DL-Met	0.16	0.12	0.07	0.08	0.11	0.13	0.11
L-Thr	0.11	0.02	0.08	0.03	0.11	0.10	0.09
L-Val	0.02	–	–	–	0.02	–	–
Vitamin mineral premix ^2^	0.15	0.15	0.15	0.15	0.15	0.15	0.15

^1^ Phase-1 diets were fed from d 1 to 14 post-weaning; phase-2 diets were fed from d 15 to 28 post-weaning; and the phase 3 diet was fed from d 29 to 42 post-weaning. ^2^ The vitamin-micromineral premix provided the following quantities of vitamins and microminerals per kg of complete diet: vitamin A as retinyl acetate, 11,136 mg; vitamin D_3_ as cholecalciferol, 2208 mg; vitamin E as DL alpha-tocopheryl acetate, 66.0 mg; vitamin K as menadione dimethylprimidinol bisulfite, 1.42 mg; thiamin as thiamine mononitrate, 0.24 mg; riboflavin, 6.59 mg; pyridoxine as pyridoxine hydrochloride, 0.24 mg; vitamin B_12_, 0.03 mg; D-pantothenic acid as D-calcium pantothenate, 23.5 mg; niacin, 44.1 mg; folic acid, 1.59 mg; biotin, 0.44 mg; Cu, 20.0 mg as copper sulfate and copper chloride; Fe, 126.0 mg as ferrous sulfate; I, 1.26 mg as ethylenediamine dihydriodide; Mn, 60.2 mg as manganese sulfate; Se, 0.30 mg as sodium selenite and selenium yeast; and Zn, 125.1 mg as zinc sulfate.

**Table 2:** Analyzed nutrient composition of experimental diets (as-fed basis) ^1^.

Item	Phase 1		Phase 2				
	Low Crude Protein		Low Crude Protein		Normal Crude Protein		Phase 3
Spray-Dried Plasma, %:	–	6.0	–	2.5	–	2.5	–
Dry matter, %	88.96	89.61	88.75	88.31	89.13	88.6	87.49
Crude protein, %	18.02	18.24	17.77	17.70	19.60	19.48	20.11
Ash, %	5.50	5.38	5.24	5.62	5.64	5.89	4.59
Acid hydrolyzed ether extract, %	4.17	4.53	5.43	5.71	4.03	4.92	5.57
Gross energy, kcal/kg	3947	4029	4001	4034	4019	4012	3999
Starch, %	32.80	34.57	34.34	32.54	31.15	28.79	36.17
Minerals, %							
Ca	0.90	0.93	0.75	0.79	0.73	0.88	0.89
P	0.63	0.68	0.64	0.70	0.66	0.70	0.71
K	0.84	1.13	0.96	0.98	1.15	1.19	1.15
Mg	0.11	0.14	0.14	0.16	0.15	0.16	0.20
Na	0.23	0.69	0.25	0.33	0.29	0.43	0.08
Indispensable amino acids, %							
Arg	1.01	0.97	1.04	1.02	1.15	1.15	1.22
His	0.43	0.46	0.44	0.45	0.48	0.49	0.50
Ile	0.80	0.77	0.80	0.77	0.91	0.87	0.87
Leu	1.52	1.66	1.51	1.56	1.64	1.69	1.64
Lys	1.29	1.37	1.23	1.24	1.50	1.59	1.32
Met	0.36	0.36	0.35	0.30	0.37	0.39	0.41
Phe	0.85	0.90	0.88	0.89	0.96	0.98	1.01
Thr	0.79	0.88	0.75	0.78	0.85	0.90	0.79
Trp	0.22	0.25	0.21	0.27	0.26	0.27	0.25
Val	0.89	1.02	0.87	0.92	0.99	1.02	0.96
Total	8.16	8.64	8.08	8.20	9.11	9.35	8.97
Dispensable amino acids, %							
Ala	0.85	0.90	0.86	0.88	0.92	0.95	0.95
Asp	1.75	1.77	1.72	1.72	1.97	1.97	1.98
Cys	0.26	0.38	0.28	0.31	0.31	0.36	0.30
Glu	3.03	2.91	3.03	2.93	3.37	3.31	3.42
Gly	0.66	0.65	0.70	0.69	0.76	0.76	0.81
Pro	0.97	1.00	0.96	0.98	1.04	1.05	1.07
Ser	0.76	0.84	0.74	0.80	0.82	0.86	0.84
Tyr	0.57	0.64	0.59	0.63	0.63	0.68	0.66
Total	8.85	9.09	8.88	8.94	9.82	9.94	10.03
Total amino acids, %	17.01	17.73	16.96	17.14	18.93	19.29	19.00

^1^ Samples were analyzed in duplicate.

**Table 3:** Analyzed nutrient composition of ingredients (as-fed basis) ^1^.

Item	Spray-Dried Plasma	Corn	Soybean Meal	Whey Powder	Soy Protein Concentrate
Dry matter, %	90.75	86.81	87.98	89.96	92.23
Crude protein, %	82.11	7.27	45.93	11.36	63.44
Ash, %	7.32	0.69	6.73	7.50	6.97
Acid hydrolyzed ether extract, %	0.36	3.67	2.30	0.31	0.74
Gross energy, kcal/kg	4847	3867	4158	3636	4415
Minerals, %					
Ca	0.07	0.01	0.33	0.61	0.38
P	0.89	0.28	0.61	0.75	0.91
K	0.12	0.30	1.81	2.55	2.37
Mg	0.02	0.08	0.24	0.14	0.36
Na	1.31	0.01	0.08	0.70	0.01
Indispensable amino acids, %					
Arg	4.53	0.35	3.08	0.26	4.57
His	2.41	0.20	1.13	0.21	1.65
Ile	2.57	0.25	2.12	0.70	3.04
Leu	7.49	0.77	3.39	1.14	4.83
Lys	7.20	0.25	2.76	0.90	4.03
Met	0.95	0.14	0.61	0.18	0.88
Phe	4.26	0.35	2.34	0.38	3.28
Thr	5.09	0.25	1.67	0.71	2.39
Trp	1.56	0.05	0.65	0.22	0.83
Val	5.67	0.34	2.19	0.67	3.14
Total	41.73	2.95	19.94	5.37	28.64
Dispensable amino acids, %					
Ala	3.84	0.50	1.88	0.54	2.58
Asp	7.89	0.48	4.85	1.14	6.89
Cys	2.62	0.15	0.62	0.26	0.90
Glu	10.84	1.20	7.85	1.89	11.33
Gly	2.77	0.30	1.85	0.24	2.43
Pro	3.97	0.56	2.12	0.63	3.03
Ser	4.74	0.31	1.97	0.47	2.74
Tyr	3.74	0.23	1.60	0.27	2.20
Total	40.41	3.73	22.74	5.44	32.10
Total amino acids, %	82.14	6.68	42.68	10.81	60.74

^1^ Samples were analyzed in duplicate.

**Table 4:** Influence of inclusion of spray-dried plasma (SDP) and concentration of crude protein (CP) in phase 1 or phase 2 diets fed to weaned pigs on growth performance ^1,2,3^.

Phase-1 SDP:	0%		6%				Pooled SEM	Contrasts ^4,5^
Phase-2 SDP:	0%	0%	0%	2.5%	0%	2.5%		
Phase-2 CP:	Low	Normal	Low	Low	Normal	Normal		
d 1 to 14								
Initial BW, kg	6.39	6.33	6.37	6.37	6.33	6.35	0.22	–
ADG, g	132	114	176	167	163	163	8.36	c **
ADFI, g	182	176	231	217	218	231	17.10	c **
G:F	0.72	0.66	0.77	0.77	0.75	0.73	0.05	c **
d 14 BW, kg	8.16	8.02	8.82	8.67	8.58	8.72	0.25	c **
d 15 to 28								
ADG, g	440	529	485	460	508	572	28.73	a *, b, c, d, e **
ADFI, g	656	696	733	720	686	763	26.69	a, b, c *, d *
G:F	0.67	0.75	0.66	0.64	0.74	0.76	0.03	e **
d 28 BW, kg	14.40	15.58	15.69	15.27	15.76	16.85	0.47	a *, c **, d *, e **
d 29 to 42								
ADG, g	670	636	693	654	631	661	19.50	a, e
ADFI, g	877	899	919	898	862	951	29.74	A
G:F	0.76	0.71	0.75	0.73	0.74	0.70	0.01	d, e *
Final BW, kg	23.11	23.79	24.74	23.75	23.94	25.45	0.65	a *, c *, d
d 1 to 42								
ADG, g	412	426	452	426	434	466	12.46	a *, c **
ADFI, g	570	588	628	610	591	648	18.16	a *, b, c **, d *
G:F	0.72	0.73	0.72	0.70	0.74	0.72	0.02	d *, e *

^1^ ADFI, average daily feed intake; ADG, average daily gain; BW, body weight; G:F, gain-to-feed ratio. ^2^ Phase-1 diets were fed from d 1 to 14 post-weaning, phase-2 diets were fed from d 15 to 28 post-weaning, and the common phase 3 diet was fed from d 29 to 42 post-weaning. ^3^ Growth performance parameters were based on 5 pigs per pen from d 1 to 28, and 4 pigs per pen from d 29 to 42. ^4^ Contrasts that were significant (*p* < 0.05) or tended (*p* < 0.10) to be significant were expressed as follows: a = interaction within phase-1 diets with 6% SDP, phase-2 SDP × phase-2 CP; b = interaction within phase-2 diets without SDP, phase-1 SDP × phase-2 CP; c = main effect of phase-1 SDP; d = main effect of phase-2 SDP; e = main effect of phase-2 CP. ^5^ No symbol = *p* < 0.10; * = *p* < 0.05; ** = *p* < 0.01.

**Table 5:** Influence of inclusion of spray-dried plasma (SDP) and concentration of crude protein (CP) in phase 1 or phase 2 diets on diarrhea scores for weaned pigs.

Phase-1 SDP:	0%		6%				Pooled SEM	Contrasts ^1,2^
Phase-2 SDP:	0%	0%	0%	2.5%	0%	2.5%		
Phase-2 CP:	Low	Normal	Low	Low	Normal	Normal		
Diarrhea score ^3^								
d 1 to 6	2.28	1.87	1.67	2.10	2.00	1.80	0.19	c
d 8 to 14	2.37	2.43	2.57	2.38	2.51	2.78	0.13	–
d 1 to 14	2.33	2.20	2.15	2.31	2.29	2.30	0.10	–
d 16 to 28	1.92	2.15	2.08	1.76	2.22	2.04	0.10	d *, e **
d 30 to 42	1.32	1.43	1.27	1.27	1.37	1.19	0.05	c *, d *
Overall	2.28	1.87	1.67	2.10	2.00	1.80	0.19	–

^1^ Contrasts that were significant (*p* < 0.05) or tended (*p* < 0.10) to be significant were expressed as follows: c = main effect of phase-1 SDP; d = main effect of phase-2 SDP; e = main effect of phase-2 CP. ^2^ No symbol = *p* < 0.10; * = *p* < 0.05; ** = *p* < 0.01. ^3^ Diarrhea score: 1 = normal feces; 2 = moist feces; 3 = mild diarrhea; 4 = severe diarrhea; 5 = watery diarrhea.

**Table 6:** Influence of inclusion of spray-dried plasma (SDP) and concentration of crude protein (CP) in phase 1 or phase 2 diets fed to weaned pigs on jejunum morphology at 28 d.

Phase-1 SDP:	0%		6%				Pooled SEM	Contrasts ^1,2^
Phase-2 SDP:	0%	0%	0%	2.5%	0%	2.5%		
Phase-2 CP:	Low	Normal	Low	Low	Normal	Normal		
Villus height, µm	509	494	520	512	526	533	22.41	–
Villus width, µm	133	135	142	143	141	135	6.63	–
Crypt depth, µm	361	339	367	349	329	384	14.02	a **
Villus height/crypt depth ratio	1.44	1.35	1.43	1.55	1.70	1.46	0.09	a *, b *, c *
Lamina propria thickness, µm	85.9	88.2	90.2	95.0	90.3	84.9	5.34	–
Villus surface area, mm	213,665	213,883	241,304	224,739	234,857	227,974	12,344.82	c

^1^ Contrasts that were significant (*p* < 0.05) or tended (*p* < 0.10) to be significant were expressed as follows: a = interaction within phase-1 diets with 6% SDP, phase-2 SDP × phase-2 CP; b = interaction within phase-2 diets without SDP, phase-1 SDP × phase-2 CP; c = main effect of phase-1 SDP. ^2^ No symbol = *p* < 0.10; * = *p* < 0.05; ** = *p* < 0.01.

**Table 7:** Influence of inclusion of spray-dried plasma (SDP) and concentration of crude protein (CP) in phase 1 or phase 2 diets fed to weaned pigs on jejunal mucosa concentrations of secretory immunoglobulin A (µg/mg of protein) and cytokines (ng/mL) ^1,2^ at 28 d.

Phase-1 SDP:	0%		6%				Pooled SEM	Contrasts ^3,4^
Phase-2 SDP:	0%	0%	0%	2.5%	0%	2.5%		
Phase-2 CP:	Low	Normal	Low	Low	Normal	Normal		
Jejunum								
sIgA	5.07	5.16	4.78	3.90	3.81	4.74	1.534	–
IL-1α	0.08	0.10	0.08	0.07	0.10	0.08	0.012	e
IL-1β	2.09	2.95	1.92	2.22	2.51	1.71	0.546	–
IL-1Ra	0.52	0.51	0.55	0.52	0.55	0.48	0.116	–
IL-2	0.08	0.08	0.07	0.09	0.08	0.07	0.015	–
IL-4	0.07	0.06	0.07	0.10	0.08	0.08	0.012	c *, d *
IL-6	0.02	0.02	0.02	0.02	0.03	0.02	0.004	–
IL-8	23.74	23.50	26.23	22.99	25.94	29.52	2.963	–
IL-10	0.05	0.04	0.04	0.05	0.05	0.04	0.004	b
IL-12	0.17	0.19	0.19	0.19	0.18	0.14	0.030	–
IL-18	24.05	29.35	22.02	23.04	25.47	26.68	6.780	e
Ileum								
sIgA	3.57	3.33	2.34	3.60	4.36	3.11	0.630	a *, b *
IL-1α	0.12	0.14	0.11	0.09	0.10	0.11	0.034	c *
IL-1β	4.33	5.02	3.42	2.47	3.50	3.33	1.662	c *, d *
IL-1Ra	0.58	0.64	0.61	0.48	0.55	0.57	0.145	–
IL-2	0.07	0.08	0.07	0.08	0.08	0.08	0.009	–
IL-4	0.06	0.04	0.05	0.07	0.06	0.07	0.010	d
IL-6	0.05	0.05	0.04	0.05	0.04	0.04	0.007	c
IL-8	19.87	32.41	27.77	22.28	32.18	32.12	4.079	e **
IL-10	0.08	0.05	0.05	0.06	0.05	0.06	0.007	b *, e
IL-12	0.30	0.34	0.27	0.26	0.25	0.27	0.036	c
IL-18	15.37	12.60	9.71	9.35	16.56	8.69	4.836	b, c, d *

^1^ IL, interleukin; IL-1Ra, interleukin-1 receptor antagonist; sIgA, secretory immunoglobulin A. ^2^ Values were Log10 transformed before analysis to obtain a normal distribution, but data are shown as back-transformed least square means. ^3^ Contrasts that were significant (*p* < 0.05) or tended (*p* < 0.10) to be significant were expressed as follows: a = interaction within phase-1 diets with 6% SDP, phase-2 SDP × phase-2 CP; b = interaction within phase-2 diets without SDP, phase-1 SDP × phase-2 CP; c = main effect of phase-1 SDP; d = main effect of phase-2 SDP; e = main effect of phase-2 CP. ^4^ No symbol = *p* < 0.10; * = *p* < 0.05; ** = *p* < 0.01.

**Table 8:** Influence of inclusion of spray-dried plasma (SDP) and concentration of crude protein (CP) in phase 1 or phase 2 diets fed to weaned pigs on blood cell counts and plasma biochemical parameters ^1^.

Phase-1 SDP:	0%		6%				Pooled SEM	Contrasts ^2,3^				Pooled SEM		
Phase-2 SDP:	0%	0%	0%	2.5%	0%	2.5%			Day				*p*-Value ^4^	
Phase-2 CP:	Low	Normal	Low	Low	Normal	Normal			7	14	28		Linear	Quadratic
White blood cells	17.42	17.78	17.11	19.22	18.38	17.65	2.29	–	12.56	25.10	16.12	2.22	<0.001	<0.001
Neutrophils	43.69	39.90	38.90	44.98	41.53	40.44	3.76	–	37.25	50.98	36.49	3.11	0.682	<0.001
Lymphocytes	49.95	54.54	54.88	48.96	50.85	51.92	3.99	–	56.07	42.00	57.47	3.36	0.433	<0.001
Plasma urea N	7.43	8.32	6.50	7.33	5.57	7.17	0.64	c *	9.36	5.92	5.88	0.47	<0.001	0.001
Albumin	2.45	2.74	2.56	2.50	2.62	2.63	0.07	b, e **	2.78	2.36	2.61	0.04	<0.001	<0.001
Total protein	4.23	4.52	4.26	4.24	4.41	4.40	0.08	e **	4.42	4.25	4.36	0.05	0.208	<0.001
AGR ^5^	1.45	1.56	1.56	1.52	1.50	1.55	0.07	–	1.74	1.28	1.55	0.05	0.002	<0.001

^1^ Units for the blood cell counts: white blood cells, ×10^3^ per µL; neutrophils, % of white blood cells; lymphocytes, % of white blood cells; plasma urea N, mg per dL; albumin, g per dL; total protein, g per dL. ^2^ Contrasts that were significant (*p* < 0.05) or tended (*p* < 0.10) to be significant were expressed as follows: b = interaction within phase-2 diets without SDP, phase-1 SDP × phase-2 CP; c = main effect of phase-1 SDP; e = main effect of phase-2 CP. ^3^ No symbol = *p* < 0.10; * = *p* < 0.05; ** = *p* < 0.01. ^4^ *p*-values were calculated to test the linear and quadratic effects of day. ^5^ AGR, albumin-to-globulin ratio.

**Table 9:** Influence of inclusion of spray-dried plasma (SDP) and concentration of crude protein (CP) in phase 1 or phase 2 diets fed to weaned pigs on concentrations of plasma amino acids (AA; µM/mL).

Phase-1 SDP:	0%		6%				Pooled SEM	Contrasts ^1,2^				Pooled SEM		
Phase-2 SDP:	0%	0%	0%	2.5%	0%	2.5%			Day				*p*-Value ^3^	
Phase-2 CP:	Low	Normal	Low	Low	Normal	Normal			7	14	28		Linear	Quadratic
Indispensable AA														
Arg	70	76	64	69	70	66	21.34	–	42	54	111	20.98	<0.001	<0.001
His	33	31	30	32	31	28	6.66	–	33	30	30	6.43	0.095	0.246
Ile	234	230	170	169	167	155	42.29	c **, d **	166	126	271	42.08	<0.001	<0.001
Leu	248	232	238	240	242	231	45.96	–	214	206	295	45.40	<0.001	<0.001
Lys	425	394	452	463	461	447	49.99	c **	385	394	542	48.26	<0.001	<0.001
Met	97	88	82	92	92	82	14.58	a, b	100	71	95	14.20	0.453	<0.001
Phe	138	133	134	141	135	126	13.54	–	121	124	157	13.27	<0.001	0.010
Thr	257	298	321	290	328	307	32.04	–	209	238	454	22.23	<0.001	<0.001
Trp	43	46	55	52	57	57	11.94	c **	44	37	75	11.87	<0.001	<0.001
Val	255	276	315	333	330	302	62.22	c **	303	274	329	60.81	0.157	0.010
Total	1818	1783	1862	1896	1967	1797	299.4	–	1622	1572	2368	294.5	<0.001	<0.001
Dispensable AA														
Ala	2340	2114	2222	2083	2118	2140	371.6	–	1908	2045	2556	359.3	<0.001	0.133
Asn	55	58	54	55	61	58	7.31	e	32	46	93	7.06	<0.001	<0.001
Asp	109	109	109	113	112	111	6.16	–	115	107	109	5.89	0.022	0.021
Cys	9	9	10	9	9	9	0.45	–	9	9	10	0.31	0.152	0.072
Gln	299	286	311	324	324	313	41.87	c *	264	281	383	40.82	<0.001	0.002
Glu	220	237	256	208	213	223	56.05	a, b *	195	256	227	54.91	0.017	<0.001
Gly	1117	1076	1253	1072	1212	1134	235.0	–	1056	940	1436	231.1	<0.001	<0.001
Pro	323	308	330	320	328	312	53.62	–	262	271	428	52.94	<0.001	<0.001
Ser	100	93	114	99	106	108	23.19	c *	76	85	148	22.96	<0.001	<0.001
Tyr	126	132	148	141	155	152	26.21	c **	98	114	215	25.74	<0.001	<0.001
Total	4707	4426	4808	4408	4729	4558	814.1	–	4041	4165	5612	803.6	<0.001	<0.001
Total AA	6525	6243	6668	6308	6696	6358	1104.4	–	5681	5737	7981	1092.4	<0.001	<0.001
Ornithine	53	57	53	53	59	56	7.12	e	30	44	91	6.87	<0.001	<0.001

^1^ Contrasts that were significant (*p* < 0.05) or tended (*p* < 0.10) to be significant were expressed as follows: a = interaction within phase-1 diets with 6% SDP, phase-2 SDP × phase-2 CP; b = interaction within phase-2 diets without SDP, phase-1 SDP × phase-2 CP; c = main effect of phase-1 SDP; d = main effect of phase-2 SDP; e = main effect of phase-2 CP. ^2^ No symbol = *p* < 0.10; * = *p* < 0.05; ** = *p* < 0.01. ^3^ *p*-values were calculated to test the linear and quadratic effects of day.

## Data Availability

The data supporting the conclusions of this article will be made available by the authors upon request.
